# Portable sensor for the detection of picric acid using fluorescent carbon dot embedded PVA film

**DOI:** 10.1039/d5ra08207b

**Published:** 2025-12-15

**Authors:** S. Priya, E. Karthikeyan, C. P. Reshmi, Princy Deni Raju, Chettiyam Veettil Suneesh, Kulangara Sandeep, A. R. Ramesh

**Affiliations:** a Department of Chemistry, Government Victoria College, Research Center Under University of Calicut Palakkad 678001 India aroramesh@gvc.ac.in; b Department of Chemistry, University of Kerala Kariavattom Campus Thiruvananthapuram-695581 India

## Abstract

The current work emphasizes the development of a carbon dot incorporated portable polymer film for the sensitive and selective detection of picric acid (PA) in aqueous solutions. Nitrogen doped fluorescent carbon dots (WCD) were synthesized from white curcumin *via* hydrothermal method and characterized using HRTEM, FTIR, XRD, Raman and XPS. This promising scaffold can detect picric acid (PA) *via* fluorescence quenching, in nanomolar range, with a limit of detection (LOD) of 4.55 ppb (19.9 nM), demonstrating sensitivity far below the permissible limit of 0.5 ppm in drinking water. The quenching mechanism is rationalized by a cumulative effect of the inner filter effect and other secondary interactions. Successful incorporation of WCD into the polymer polyvinyl alcohol (PVA) matrix resulted in a fluorescent WCD/PVA film, and acts as a promising candidate for portable, real-time environmental sensing applications. The fluorescence studies on the WCD/PVA film affirm its practicability for on-site sensing by exhibiting fluorescence quenching in 25 nM PA solution which is near to the LOD of WCD solution.

## Abstract 2

The current work emphasizes the development of a carbon dot incorporated portable polymer film for the sensitive and selective detection of picric acid (PA) in aqueous solutions.

## Graphical abstract

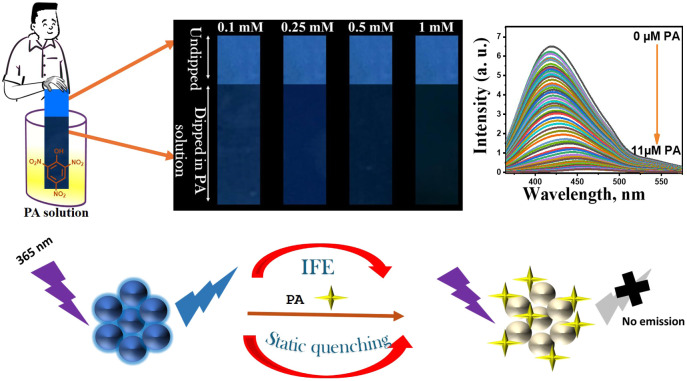

## Introduction

Among the nitroaromatic compounds, picric acid (PA) has long been used as a key ingredient in dyes, etching agents, matches, explosives and pharmaceuticals.^1^ The three nitro groups attached to the benzene ring and high sensitivity towards shock and heat intensify the explosive nature of PA.^2^ The frequent discharge of PA into water bodies and soil from various industries and factories has exacerbated its toxicity due to its high aqueous solubility.^3^ This major pollutant can cause potential health hazards, and damage kidneys, liver, blood cells and other internal organs, imbalancing the metabolism and life cycle.^4^ Even very low concentrations (permissible limit of <0.5 ppm in drinking water and permissible daily intake of <37 µg) can be lethal^5^ to the ecosystem, and it is crucial to develop an analytical probe for the selective and sensitive detection of PA.

Carbon dots are zero dimensional quasi-spherical carbonaceous materials with size less than 10 nm, exhibiting excellent fluorescent properties.^6^ This promising scaffold exhibits tunable fluorescence with resistance to photobleaching and can be employed in sensing,^7,8^ catalysis,^9^ anti-counterfeiting,^10^ bio-imaging,^7^ and other biological studies.^11^ To date, advancements in the selection of precursors from green natural sources,^12,13^ biomass, waste byproducts^14^ and chemicals^15^ are appreciable; however, the challenge still exists. Medicinal and non-medicinal herbs, invasive species, and fruit pomaces have become the precursors for synthesizing CDs with versatile applications.^16^

The photoluminescent (PL) property of CDs has gained significant attention in the scientific world, which is explained by surface functional groups, core state, and quantum confinement effects.^17^ The presence of inherent surface functional groups^18^ and those developed by heteroatom doping^1,19^ shall betterment the fluorescent properties of carbon dots. In recent studies, Mate *et al.*, synthesized TF-CD from *Thevetia* flowers senses PA with a limit of detection of 104 nM and a PVDF-based polymer film for the real time analysis of PA.^20^ Mahto *et al.*, synthesized NCDs from malic acid and urea *via* a microwave-assisted pyrolysis method and demonstrated PA detection using a molecular fluorophore based quenching mechanism.^21^ It is noteworthy that the fluorescence quenching in most of these works is not wholly contributed by a single mechanism, but a combined effect of certain primary and secondary processes.

White curcumin, scientifically known as *Curcuma zedoaria*, belongs to the Zingiberaceae family, rich in various sesquiterpenes, curcumenols, and phytosterols.^22^ It has been used in the *in vitro* and *in vivo* test models for its biological actions and reported that many of these plant parts exhibit excellent pharmacological effects. Herein we have synthesized carbon dots from white curcumin *via* hydrothermal method. Their bright blue luminescence property was utilized to selectively detect picric acid in aqueous solutions by fluorescence quenching. Further, these CDs were incorporated in PVA film to serve as a portable sensor.

## Results and discussion

**Fig. 1:**
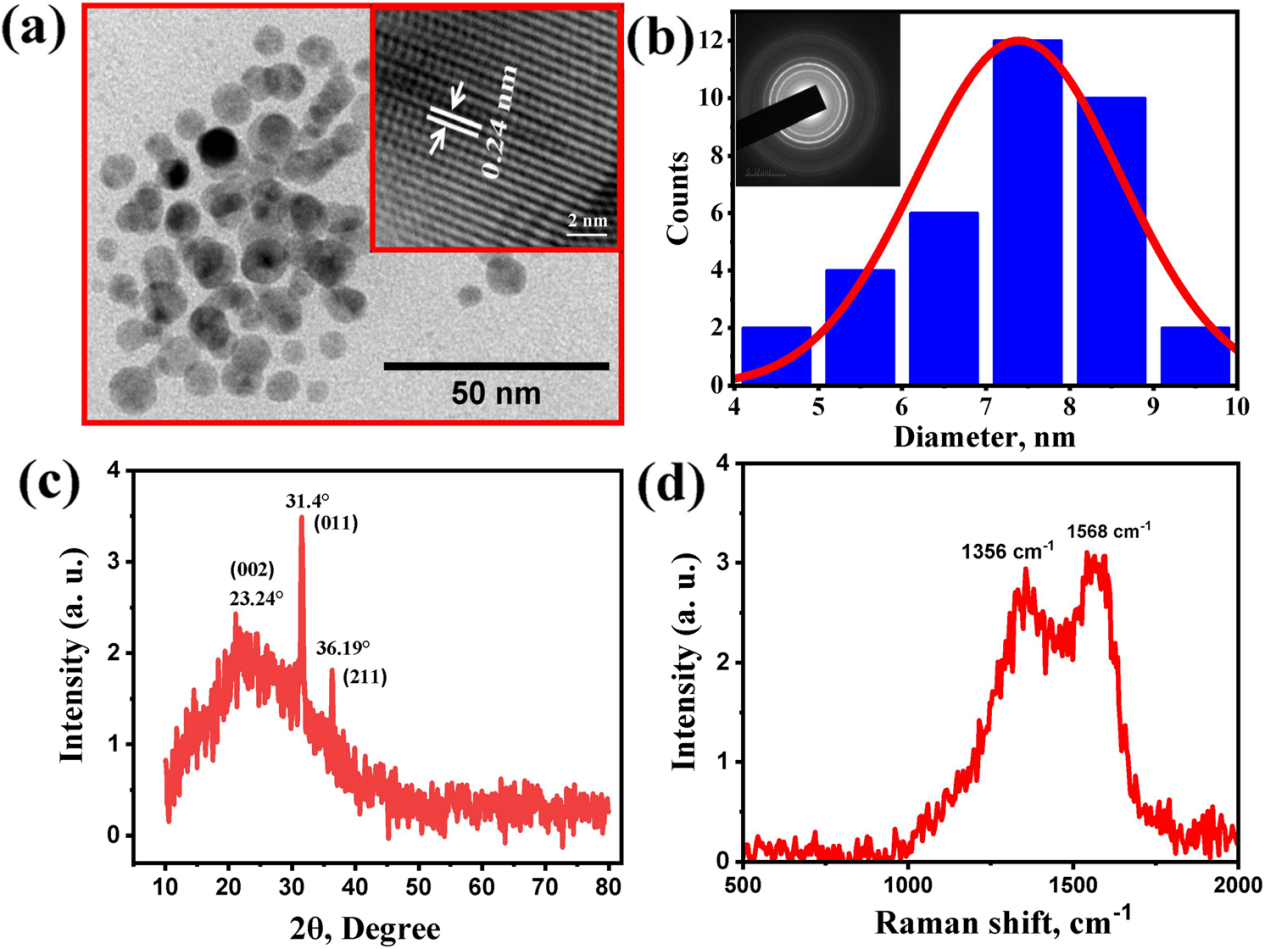
(a) TEM image of white curcumin derived carbon dots (WCD) showing nearly spherical morphology with uniform distribution; inset highlights high-resolution lattice fringes. (b) Histogram illustrating particle size distribution of WCD ranging from 4–10 nm with an average of 7.39 nm; inset shows the SAED pattern. (c) XRD pattern displaying a broad low-intensity peak at 2*θ* ≈ 23.24° (002), along with sharper peaks. (d) Raman spectrum exhibiting distinct D and G bands.

The surface morphology and characterizations of synthesized WCDs were performed using HRTEM, XRD, FTIR and XPS techniques. The HRTEM image (Fig. 1(a)) reveals nearly spherical morphology of the as synthesized WCD exhibiting partial crystalline nature, evident from the selected area electron diffraction (SAED) pattern (Fig. 1(b)). The appearance of bright circular diffraction rings in the SAED pattern shows a degree of partial crystallinity within the carbon dot structure.^23^ The histogram derived from the TEM images shows the particle size distribution of WCD ranging from 4–10 nm with an average size of 7.39 nm and interlayer spacing (*d* value) of 0.24 nm, which is in agreement with that of aromatic or graphitic carbon.^24^ A broad diffraction peak with low intensity in the XRD spectrum (Fig. 1(c)) centered at 2*θ* value of 23.24° (002) supports the predominantly amorphous nature^23,25^ and disorders^26^ in WCD. At the same time, a sharp high intense peak at 31.4° (011), and a low intense sharp peak at 36.19° (211) demonstrates the localized crystalline arrangement within the carbon dot structure possibly induced by nitrogen doping in the carbon dots, consistent with partial crystallinity observed in the SAED pattern and with the previous reports.^24,27,28^

Raman spectroscopy provides details regarding the degree of graphitization of carbon dots, and in the case of WCD two characteristic peaks were observed at 1356 cm^−1^ and 1568 cm^−1^ characteristic for the D band and G band respectively (Fig. 1(d)). The D band is associated with the structural defects present due to the sp^3^ hybridized amorphous carbons and the G band explains the graphene layer formation by the sp^2^ hybrid carbons.^29^ The *I*_D_/*I*_G_ ratio is found to be 0.88, a greater intensity of G band indicates the presence of a large number of sp^2^ carbons with fewer sp^3^ carbons in the synthesized WCD.^30^ Surface functionalization makes the carbon dot an eminent candidate for broad applications and hence, the surface functional groups can be characterized by FTIR spectroscopy. Fig. S1 shows the FTIR spectra of WCD and raw compound, where WCD shows peaks at 3366 cm^−1^, 2850 cm^−1^, 1634 cm^−1^, 1053 cm^−1^ and 868 cm^−1^ which correspond to NH/OH, C–H, C=C/C=O, C–O and C–N vibrational modes respectively.^24,26^

**Fig. 2:**
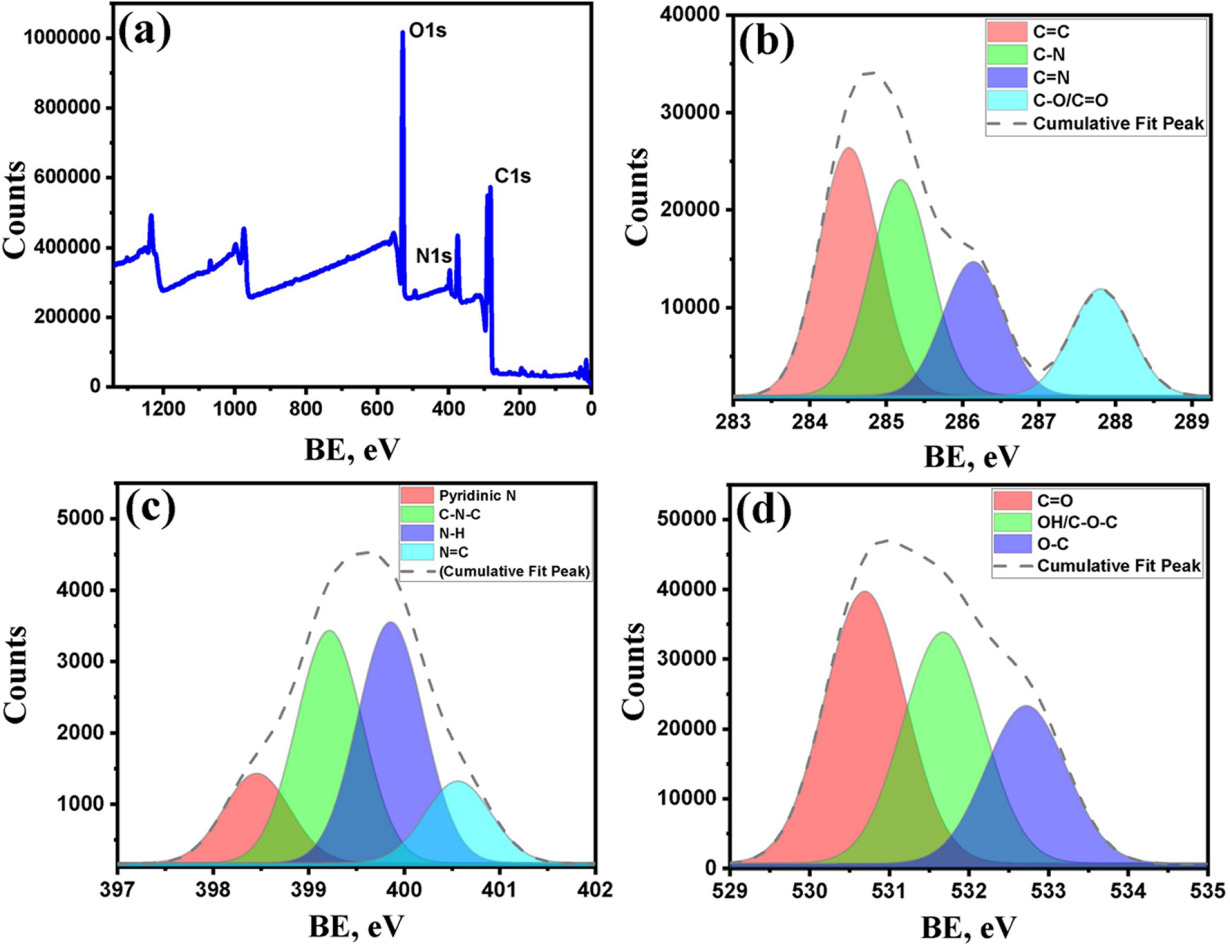
XPS analysis of WCD. (a) Wide-scan survey spectrum showing major peaks of C 1s, N 1s and O 1s, confirming the presence of carbon, nitrogen, and oxygen elements. (b) High-resolution C 1s spectrum deconvoluted into four components corresponding to C=C, C–N, C=N and C–O/C=O. (c) N 1s deconvoluted spectrum displaying peaks assigned to pyridinic-N, N–H, C–N–C, and N=C functionalities. (d) O 1s spectrum showing peaks attributed to C=O, OH/C–O–C, and O–C bonds, respectively.

Further evidence for the chemical composition and functionalities was obtained by performing X-ray photoelectron spectroscopy (XPS). Fig. 2(a) shows the XPS survey spectrum of WCD having three distinct peaks at 284.4 eV, 399.1 eV and 530.5 eV which corresponds to C 1s (58.73%), N 1s (5.31%) and O 1s (35.96%) respectively. The high resolution deconvoluted spectra of C 1s (Fig. 2(b)) shows four distinct peaks at a binding energy of 284.5 eV, 285.2 eV, 286.15 eV, and 287.83 eV, corresponding to C=C, C–N, C=N and C–O/C=O respectively. Further, the deconvoluted spectra of N 1s (Fig. 2(c)) occurs at a binding energy of 398.46 eV, 399.23 eV, 399.86 eV and 400.58 eV attributed to pyridinic-N, N–H, C–N–C and N=C respectively and the high-resolution spectrum of O 1s (Fig. 2(d)) at 530.69 eV, 531.70 eV and 532.72 eV corresponding to C=O, OH/C–O–C and O–C bonds respectively.

Fig. S2(a) represents the combined spectra showing the UV-vis absorption, excitation and emission profiles of WCD. The UV-vis absorption spectrum of WCD (green line in Fig. S2(a)) exhibits a prominent peak at 270 nm and a broad shoulder extending from 295 to 325 nm. The peak at 270 nm is attributed to π–π* electronic transitions of C=C bonds in the sp^2^-hybridized carbon atoms, while the shoulder corresponds to n–π* transitions of C=O bonds present in carboxylic and carbonyl functional groups on the surface of the carbon dots.^31^ Fig. S2(b) demonstrates the excitation wavelength dependent emission behaviour of WCD. As the excitation wavelength increased from 270 nm to 320 nm, there was no considerable change in the emission wavelength and it centered around 410 nm. When the excitation wavelength was varied from 330 nm to 345 nm the emission wavelength underwent a slight red shift from 411 nm to 419 nm, and maximum emission intensity was obtained at an excitation of 345 nm. As the excitation wavelength is increased from 345 nm to 450 nm, a bathochromic shift was observed over an emission range of 419 nm to 500 nm with a steady decrease in emission intensity. The excitation dependent emission of WCD can be mainly attributed to the presence of nitrogen and oxygen containing functional groups like NH_2_, OH, and COOH on the surface of carbon dots^32^ which influences the degree of surface oxidation and can reduce the HOMO–LUMO energy gap causing a significant red shift in emission.^33,34^ Another possible reason could be heteroatom doping, which creates different surface emissive traps of variable energies due to surface defects.^35^ The absolute quantum yield of WCD was measured using an integrating sphere setup, and was found to be 6.25%, confirming the moderate fluorescence efficiency of the synthesized carbon dots.

pH plays a significant role in areas such as environmental monitoring, industries and agriculture, hence it is relevant to conduct pH monitoring in the research field and real-world applications.^36^ The pH sensitivity studies of WCD were conducted by varying the pH between 2 and 13 at an excitation wavelength of 345 nm (Fig. S3(a)). The emission intensity of WCD is found to be pH sensitive. An increase in emission intensity at 419 nm was observed from pH 2 to 4 and decreased at higher pH. Fluorescence intensity remained nearly constant between a pH range of 5–9, enhancing its application in biological systems.^37^ The decrease in PL intensity at higher pH may be due to the deprotonation of surface functional groups that disrupt the electronic energy levels in carbon dots causing nonradiative recombination.^38,39^ The surface functional groups present on the carbon dots may undergo deprotonation and protonation which in turn affects the energy levels causing a pH sensitive fluorescence.^40^ The effect of ionic strength on the fluorescent intensity of WCD was studied in the presence of various concentrations of NaCl. The PL intensity of WCD in NaCl at varying concentrations from 0.2 M to 2 M is shown in Fig. S3(b). Only a negligible change in PL intensity was observed, confirming the excellent ionic stability of WCD and suggesting that electrostatic screening by salt ions does not significantly affect its surface emissive states.

**Fig. 3:**
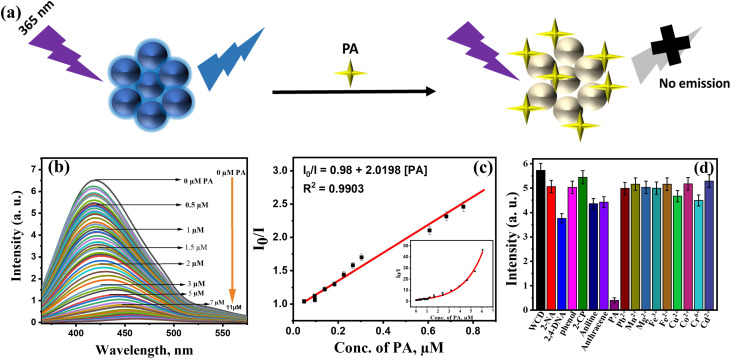
(a) Schematic illustration of the fluorescence quenching mechanism of WCD in the presence of picric acid. (b) Fluorescence spectra showing gradual quenching of WCD emission with increasing PA concentration from 0 to 11 µM. (c) Linear Stern–Volmer plot of *I*_0_/*I versus* low concentration range of PA (0–0.8 µM). Inset: 0–6 µM. (d) Selectivity study comparing the fluorescence response of WCD to PA against other nitroaromatic compounds, non-nitroaromatics, and metal ions.

WCD can be effectively employed as a sensor at room temperature for the selective and sensitive detection of PA with LOD in the nanomolar range. Upon the incremental addition of PA (0–11 µM), a progressive decrease in fluorescence intensity of WCD was observed, indicating a strong interaction between the nitroaromatic analyte and the carbon dots. Fig. 3(a) illustrates the schematic representation of quenching of WCD in the presence of PA and Fig. 3(b) shows the gradual decrease in fluorescence intensity with increased concentration of PA.

The fluorescence quenching behavior of WCD in the presence of increasing concentrations of PA was analyzed using the Stern–Volmer relationship (Fig. 3(c)). A linear correlation between the fluorescence intensity ratio (*I*_0_/*I*) and PA concentration was observed in the low concentration range of 0–0.8 µM, described by the equation 
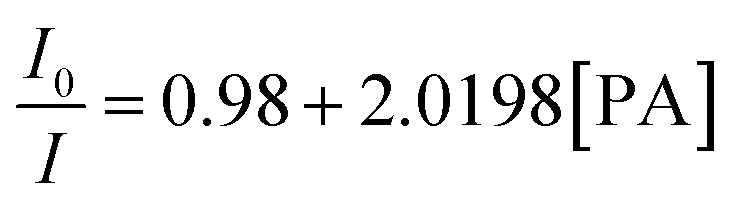
 with a correlation coefficient (*R*^2^) of 0.9903. The Stern–Volmer constant (*K*_sv_) was calculated to be 2.02 × 10^5^ M^−1^, suggesting a strong quenching interaction between WCD and PA molecules. The limit of detection (LOD) was calculated from Fig. 3(c) using the equation 
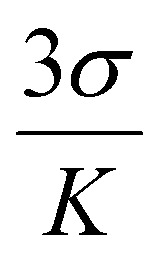
 and limit of quantification (LOQ) using the equation 
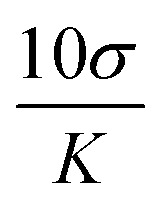
, where *σ* is the standard deviation and *K* is the slope of the calibration curve. The limit of detection (LOD) and limit of quantification were found to be 19.9 nM and 66.5 nM respectively, demonstrating the high sensitivity of WCD towards trace level PA detection.

At higher concentrations of PA (inset, Fig. 3(c)), a deviation from linearity was noted, indicating the involvement of additional non-linear quenching processes. Such deviations are often attributed to secondary interactions, such as ground-state complex formation. The high *K*_sv_ value and excellent linearity at low PA concentrations confirm the high sensitivity of WCD towards PA, enabling its detection down to nanomolar levels. This strong quenching efficiency demonstrates the suitability of WCD as an effective fluorescent probe for trace-level detection of PA in aqueous media. Table 1 compares the performance of WCD with previously reported carbon dot based PA sensors, revealing that WCD exhibits superior sensitivity and lower detection limits, demonstrating its remarkable performance among similar systems.

**Table 1:** Comparison of different carbon dot systems in PA sensing

Sl. no.	Carbon dot system	Method of synthesis	LOD (µM)	References
1	N CDs@MSN-NH_2_	Hydrothermal	0.050 µM	3
2	TF-CDs	Hydrothermal	0.244 µM	20
3	NCDs	Microwave assisted pyrolysis	0.033 µM	21
4	NS-CQDs	Hydrothermal	0.24 µM	43
5	N-CDs	Hydrothermal	0.11 µM	52
6	SGQDs	Pyrolysis	0.093 µM	53
7	CQD@gemini surfactant assembly	Microwave pyrolysis	0.0275 µM	50
8	WCD	Hydrothermal	0.019 µM	This work

A fluorescent probe should exhibit high selectivity towards a particular analyte to function effectively as a sensor. In the case of WCD, its selectivity towards PA was confirmed in the presence of interfering nitro aromatic compounds, non-nitro aromatic compounds and other metal ions such as 2,4-dinitroaniline (2,4-DNA), 2-nitro aniline (2-NA), 2-chlorophenol (2-CP), phenol, anthracene, aniline, Pb^2+^, Mn^2+^, Mg^2+^, Fe^3+^, Fe^2+^, Cu^2+^, Co^2+^, Cr^6+^ and Cd^2+^, all tested at the same concentration using the fluorescence quenching method. Fig. 3(d) demonstrates the observation that the fluorescent intensity of WCD steadily decreased in the presence of PA and no significant decrease was shown by WCD in the presence of the aforementioned compounds, which affirms the excellent selectivity of WCD towards PA detection. The pronounced selectivity of WCD towards PA can be attributed to strong electron-withdrawing nitro groups in PA, which enable efficient hydrogen bonding interactions with the surface functional groups (–OH, –NH, –COOH) of WCD, leading to preferential fluorescence quenching.

To elucidate the quenching mechanism, it is essential to examine various fluorescent quenching processes such as static quenching (SQ),^41^ dynamic quenching (DQ),^42^ Förster resonance energy transfer (FRET)^21,43^ and inner filter effect (IFE).^20,44^ SQ involves ground-state complex formation without lifetime change,^20,45,46^ while DQ and FRET typically result in a measurable decrease in fluorescence lifetime.^42,47,48^ IFE occurs when the quencher absorbs excitation or emission light, leading to intensity loss without altering excited-state dynamics.^20,49,50^

**Fig. 4:**
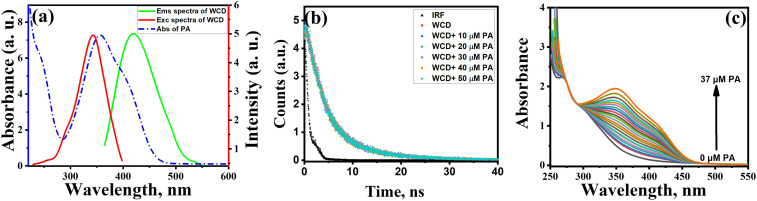
Investigation of the quenching mechanism of WCD by PA. (a) Excitation and emission spectra of WCD with the absorption spectra of PA. (b) Time-correlated single photon counting (TCSPC) lifetime decay curves of WCD in the absence and presence of PA (0–50 µM). (c) UV-vis absorption spectra of WCD and WCD–PA mixtures at increasing PA concentrations, displaying two isosbestic points at 258 and 308 nm.

On optical analysis, two key observations emerged: (1) there is a strong overlap between the excitation spectra of WCD and absorption spectra of PA which is evident from the merged excitation and emission spectra of WCD and absorption spectra of PA as shown in Fig. 4(a). PA has a prominent absorption peak at 358 nm and a broader shoulder peak around 400 nm which strongly overlaps with the excitation spectra (*λ*_ex_ = 345 nm) and partially overlaps with emission spectra (*λ*_em_ = 419 nm) of WCD. (2) TCSPC fluorescence lifetime of WCD is measured as 4.55 ns. Upon addition of 50 µM PA, it nearly remained a constant value of 4.50 ns.^51^Fig. 4(b) clearly elucidates that the fluorescence lifetime is nearly the same in the presence and absence of the quencher (PA). These findings strongly indicate that IFE is the primary cause of the quenching mechanism in WCD. Moreover, absorption spectra of WCD–PA mixtures (Fig. 4(c)) revealed isosbestic points at 258 and 308 nm, indicating specific molecular interactions. Hydrogen bonding between WCD surface groups (–OH, –NH/NH_2_, –COOH) and PA's phenolic and nitro groups likely contributes as a secondary effect, supported by a red shift in emission maximum (Fig. 3(b)).

**Fig. 5:**
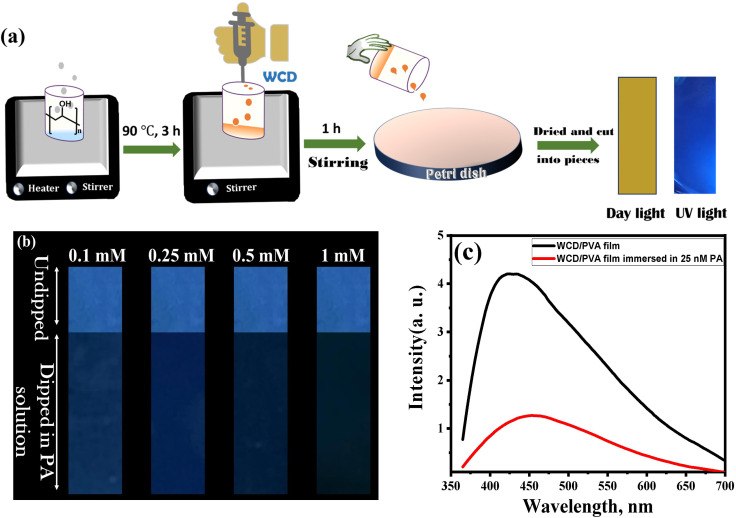
(a) Schematic representation of stepwise preparation of the WCD/PVA composite film for portable sensing applications. (b) Photographs of WCD/PVA film strips exposed to increasing PA concentrations (0.1–1 mM), captured under a 365 nm UV lamp using a smartphone camera. Three-fourths of each strip was immersed in PA solution, leaving one-fourth as an internal reference. (c) Solid-state fluorescence spectra of WCD/PVA film immersed in 25 nM PA solution, showing a progressive decrease in emission intensity.

To demonstrate the practical applicability of the developed system, a polyvinyl alcohol (PVA)-based polymer film embedded with WCDs (WCD/PVA) was fabricated (Fig. 5(a)) and employed as a portable sensor for the detection of PA. This film exhibited bright blue fluorescence under UV light, making it suitable for the detection of PA *via* fluorescence quenching. As a control, the native PVA film (without WCD) was also tested and showed no fluorescence. The visual appearance of fluorescence (Fig. 5(a)), SEM images (Fig. S4) and the fluorescence spectrum (Fig. 5(c)) of WCD/PVA film confirms the incorporation of WCD in PVA film. The WCD/PVA film was cut into equal pieces (size 1.5 cm × 6 cm) and 4 cm of each film was dipped into the PA solutions of different concentrations, then air dried at ambient conditions. The decrease in fluorescence intensity was visually monitored. Fig. 5(b) depicts the photographs of WCD/PVA–PA systems with variable concentrations of PA ranging from 0.1 mM to 1 mM taken using a smartphone camera. In each case, the three fourths of a film strip is immersed in PA solution and compares the fluorescence intensity with the undipped one fourth portion. The fluorescence quenching of WCD/PVA film was further studied by fluorescence spectroscopy. The WCD/PVA film was dipped in 25 nM PA solution and the emission spectra were recorded and a considerable decrease in the fluorescence intensity was observed. Fig. 5(c) shows the fluorescence spectra of WCD/PVA film immersed in 25 nM PA and it affirms that WCD/PVA film can be used for the detection of PA in the nanomolar range. These findings establish that the as-prepared WCD/PVA polymer film serves as a simple, portable, and highly efficient fluorescent sensor for the real-time, selective detection of picric acid in aqueous media.

## Conclusion

Nitrogen-doped carbon dots (WCDs) were successfully synthesized from white curcumin through a facile hydrothermal route and exhibited bright blue fluorescence, excellent water dispersibility, and remarkable stability against pH and ionic strength variations. Comprehensive characterization using HRTEM, XRD, FTIR, Raman, and XPS confirmed the partially crystalline, nitrogen-enriched carbon structure. The synthesized WCDs demonstrated highly sensitive and selective detection of picric acid (PA) *via* fluorescence quenching, achieving a limit of detection of 19.9 nM (4.55 ppb) significantly below the permissible limit of 0.5 ppm in drinking water. The quenching mechanism was attributed primarily to the inner filter effect (IFE), supported by secondary interactions. Furthermore, the incorporation of WCDs into a polyvinyl alcohol (PVA) matrix yielded a flexible, transparent, and portable WCD/PVA film that retained the excellent optical properties of WCDs. The film enabled real-time visual detection of PA even at nanomolar levels, underscoring its potential for on-site environmental monitoring and practical sensing applications. This work introduces a green, low-cost, and effective approach for the fabrication of portable fluorescent sensors, advancing the application of biogenic carbon dots in environmental and analytical chemistry.

## Materials and methods

### Materials

Fresh white curcumin was collected from the local regions of the Palakkad district of Kerala, India. All other chemicals used were of analytical grade and used without further purification. PA (98%) was purchased from Atom Scientific and urea (>95%) was supplied by Isochem Chemicals. For all the experimental purposes and characterization, deionised water was used.

### Preparation of WCD

White curcumin collected from the local regions was thoroughly washed with deionized water. It was dried in the shade and crushed into powder. 3 g of white curcumin powder was weighed and transferred into a beaker containing 50 mL of deionized water maintained at 100 °C and stirred for 30 min. The solution was further filtered using Whatman no. 40 filter paper and centrifuged at 10000 rpm for 30 min. The obtained clear solution was mixed with 0.2 g of urea and sonicated for 10 min to get a homogeneous solution. The solution was then transferred to a 100 mL Teflon-lined autoclave and underwent hydrothermal treatment at 180 °C for 5 h. Later, the reactor was cooled to room temperature and the WCD formed was filtered through Whatman no. 40 filter paper and centrifuged at 10 000 rpm for 30 min. The aqueous solution of WCD was further purified by dialysis with a dialysis bag of 14 kDa for 24 h and the purified WCD (0.02 g mL^−1^) stored at 4 °C for further characterizations.

### Preparation of WCD/PVA polymer film

In the preparation of WCD/PVA polymer film, PVA acts as a good template for the incorporation of WCD. 2 g of PVA grains were added to 20 mL of deionized water and heated to 90 °C for 3 h. This resulted in a homogenous solution. The heating was stopped and stirring continued for an additional 1 h. When it reached room temperature, 2 mL (0.02 g mL^−1^) of WCD was added and stirring continued for one more hour. The resulting pale brown solution is then poured into a clean Petri dish and allowed to dry at ambient room temperature (28 ± 2 °C) under dust free conditions for ∼48 h until complete solvent evaporates. After drying, WCD/PVA (2 w/w%) film was peeled off from the Petri dish and cut into desired shapes.

### Preparation of solutions

#### Preparation of PA solution and procedure for detecting PA

The stock solutions of PA and other analytes were prepared in ultrapure water. First, a stock solution of PA (1 mM, 10 mL) was prepared. Different concentrations of PA ranging from 0.01 mM to 1 mM (10 µL each) were added to the quartz cuvette containing deionized water and WCD (100 µL) with a final volume of 2 mL. After the addition of each concentration of PA, the fluorescence measurements were carried out immediately in the emission range of 300–600 nm (*λ*_exc_ = 345 nm) at room temperature. The quenching experiments were performed by sequentially adding known aliquots of a standard picric acid solution into a fixed volume (100 µL) of WCD and making the final volume to 2 mL by adding deionised water under stirring, ensuring uniform mixing. Each addition was made using a calibrated micropipette with an accuracy of ±0.5 µL. To account for possible pipetting errors, three independent titration experiments were carried out under identical conditions, and the average values were reported. Controlled experiments were carried out in the absence of PA to validate the dilution effects and no significant change in fluorescence intensity was observed.

#### Preparation of NaCl stock solution and ionic studies

To study the effect of ionic strength, 2 M NaCl stock solution was prepared in deionized water. For the study, 100 µL of WCD solution was added to different concentrations of NaCl (0.2–2.0 M) prepared in deionized water to make a final cuvette volume of 2 mL. Fluorescence measurements were recorded immediately after mixing (within 2 min) without any additional incubation, to avoid variations due to time-dependent aggregation.

### Selectivity and interference studies

Selectivity in the detection of PA was analyzed by performing fluorescence measurements in the presence of various nitro aromatic compounds, non-nitro aromatic compounds and some metal ions. Stock solutions (1 mM, 10 mL) of all the analytes were prepared and the same concentration (10 µM) was added to 100 µL WCD solution and the final volume was made up to 2 mL by adding ethanol–water (1 : 1, v/v) mixture. Fluorescence emission spectra of various analytes were recorded in the emission range of 300 to 600 nm (*λ*_ex_ = 345 nm).

### Calculations of quantum yield (QY)

The absolute quantum yield of WCD was measured by the direct excitation method which records the scatter and emission of the sample being directly excited by the radiation from the excitation monochromator only. The absolute fluorescence quantum yield by the direct method is calculated as
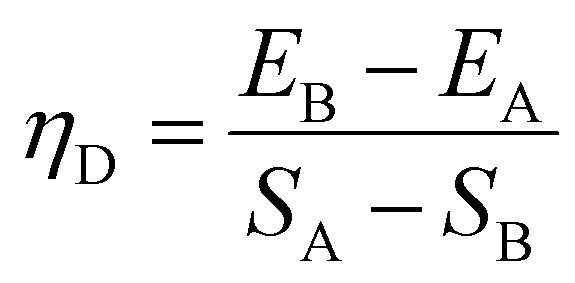
*E*_A_, *E*_B_, *S*_A_ and *S*_B_ are integrals of the scan and *η*_D_ is the absolute QY. The selection of integral regions, calculation of integral scans and the final QY values are all carried out by the quantum yield wizard provided with the FLUORACLE software.

### Instrumental methods

The UV-vis absorption spectra were recorded using a Shimadzu double beam spectrophotometer (UV-vis-NIR). JASCO FP8300 fluorescence spectrometer was used for PL studies. The X-ray diffraction (XRD) patterns were recorded using a Rigaku Miniflex-II diffractometer using Cu Kα radiation in the scan range of 2*θ* 20–80°. JEOL-JEM 2100 high resolution transmission electron microscope was used for particle size determination and morphological studies. Carbon coated copper grid was used for the sample preparation for TEM analysis and ImageJ software was used for particle size analysis. Fourier transform infrared (FTIR) spectra were recorded using the Shimadzu IR affinity model 1S instrument by the ATR method. The Raman spectrum was recorded using WiTec alpha 300, Germany spectrometer with a 532 nm laser microprobe, and the XPS analysis was performed using Thermo Scientific ESCALAB Xi^+^. The lifetime studies were conducted using Horiba-Fluoromax-4 and the fluorescence quantum yield was measured by absolute method using the FLS 1000 Edinburgh Instrument equipped with an integrating sphere. The surface morphological studies of polymer film by scanning electron microscopy (SEM) was carried out by the Zeiss Sigma 360 instrument.

## Conflicts of interest

There are no conflicts to declare.

## Supplementary Material

RA-015-D5RA08207B-s001

## Data Availability

The data supporting this article have been included as part of the supplementary information (SI). Supplementary information is available. See DOI: https://doi.org/10.1039/d5ra08207b.
