# Supplementary material for: Portable sensor for the detection of picric acid using fluorescent carbon dot embedded PVA film
Source: RSC Adv. 2025 Dec 15;15(58):50001–9. doi: 10.1039/d5ra08207b (PMC12704588; doi:10.1039/d5ra08207b)
Supplement: RA-015-D5RA08207B-s001 [file RA-015-D5RA08207B-s001.pdf]

Supplementary Information (SI) for RSC Advances.
This journal is © The Royal Society of Chemistry 2025

Supplementary Information

Portable Sensor for the Detection of Picric Acid using Fluorescent Carbon Dot Embedded PVA Film

Priya S.,[a] Karthikeyan E.,[a] Reshmi C. P.,[a] Princy Deni Raju,[b] Chettiyam Veettil Suneesh,[b] Kulangara Sandeep[a] and A. R. Ramesh[a]*

[a]*Department of Chemistry, Government Victoria College, Research Center under University of Calicut, Palakkad 678001, India.*

[b]*Department of Chemistry, University of Kerala, Kariavattom Campus, Thiruvananthapuram- 695581*

**Corresponding author:* aroramesh@gvc.ac.in

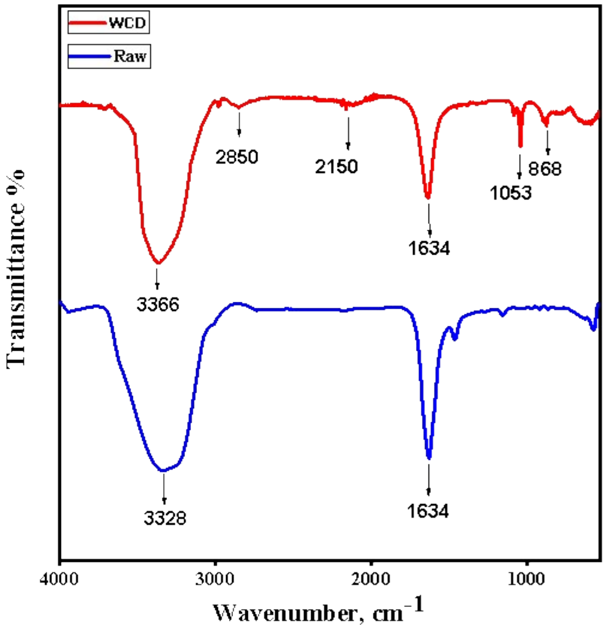

Fig. S1 FTIR spectra of white curcumin (raw) and WCD. The WCD spectrum exhibits additional bands at 3366, 2850, 2150, 1053, and 868 cm^{-1}, corresponding to NH/OH, C–H, C≡C, C–O, and C–N vibrations, respectively. These changes confirm structural modification and introduction of new functional groups upon carbonization and nitrogen doping.

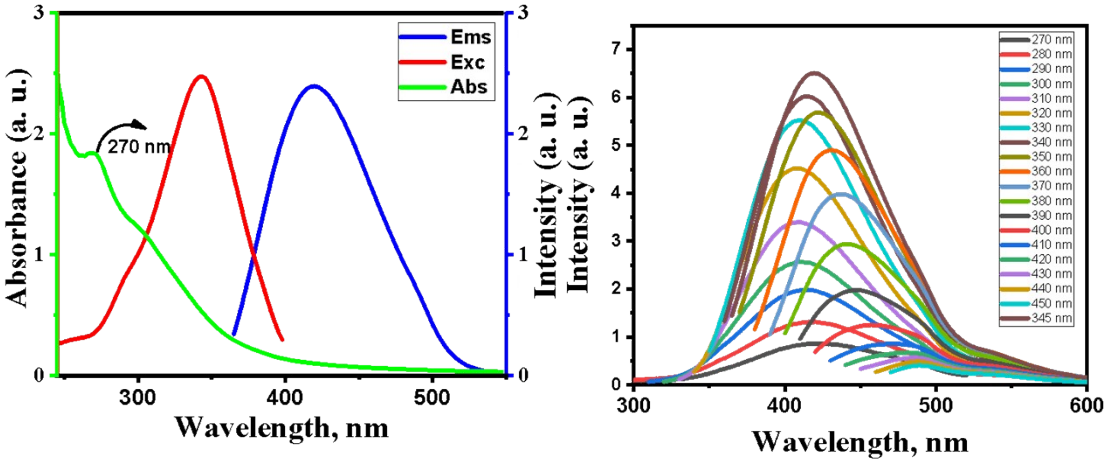

Fig. S2 Optical properties of WCD. (a) Combined UV–Vis absorption, excitation, and emission spectra. (b) Excitation-dependent emission spectra recorded across different excitation wavelengths. The emission remains centered near 410 nm at low excitations (270–320 nm), shows a slight red shift to 419 nm at 345 nm excitation, and gradually shifts up to 500 nm with decreasing intensity at higher excitations.

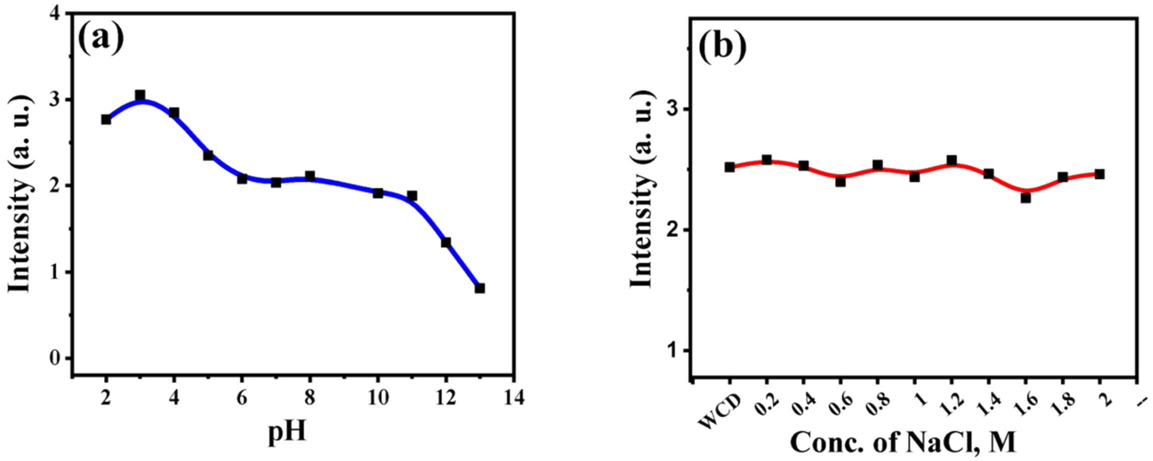

Fig. S3 pH and ionic strength studies of WCD. (a) Fluorescence intensity of WCD at pH values ranging from 2 to 13 at 345 nm excitation. (b) Effect of ionic strength on fluorescence intensity in the presence of NaCl at concentrations ranging from 0.2 M to 2 M. Only a negligible change in emission was observed, confirming strong ionic stability of WCD and suitability for biological and environmental sensing applications.

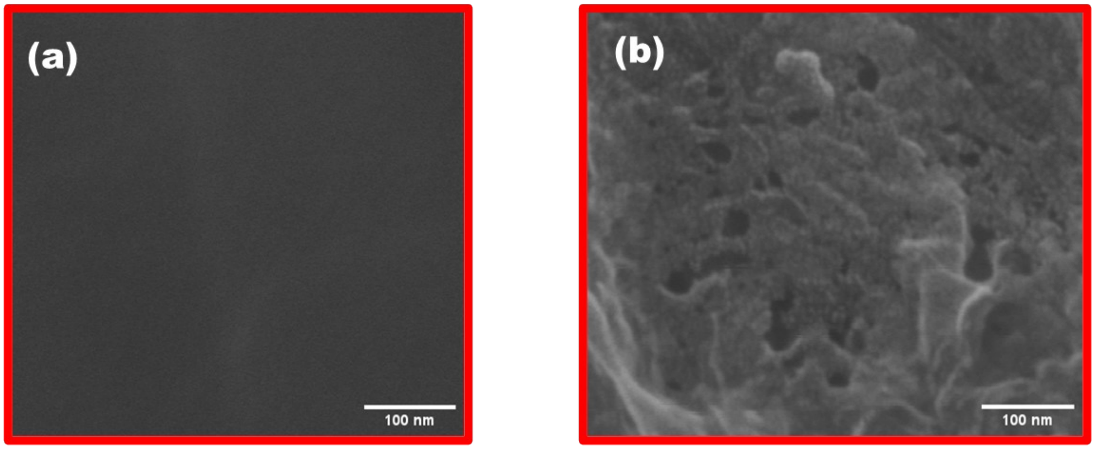

Fig. S4 Surface morphology analysis of polymer films using SEM. (a) SEM image of bare PVA film showing a relatively smooth and uniform surface morphology without any noticeable particulate features. (b) SEM image of WCD-incorporated PVA film, where the embedded carbon dots appear well-dispersed throughout the polymer matrix, leading to slight surface roughness and visible textural changes.
